# Antibiofilm efficacy of plant extracts as root canal irrigants in endodontics: a systematic literature review

**DOI:** 10.3389/fdmed.2024.1479953

**Published:** 2024-10-24

**Authors:** Jihad Diouchi, Babacar Touré, Sonia Ghoul

**Affiliations:** International Faculty of Dental Medicine, Health Sciences Research Center (CReSS), College of Health Sciences, International University of Rabat, Sala-Al Jadida, Morocco

**Keywords:** biofilm, endodontic infection, plant extract, root canal irrigant, essential oil, systematic review

## Abstract

**Background:**

To explore the antibiofilm efficacy of plant extracts against *in vitro* formed single and multispecies endodontic biofilms, in comparison to conventional root canal irrigants.

**Methods:**

PubMed, Scopus, Web of Science, and EMBASE were searched up to April 2024. Studies investigating the antibiofilm efficacy, of at least one plant extract and one conventional root canal irrigant, against endodontic biofilms were reviewed in accordance with the Preferred Reporting Items for Systematic Reviews and Meta-Analyses (PRISMA) statement guidelines. Data were extracted, and studies were critically assessed using the Joanna Briggs Institute checklist.

**Results:**

Among 78 articles, eight articles met the criteria and were eventually included in this review. One study showed a high risk of bias, six showed a moderate risk of bias, and one showed a low risk. A total of twelve plant extracts were tested for their antibiofilm efficacy against eight different single-species biofilms and one multispecies biofilm. A combination of microscopy methods and culturing techniques was used for the assessment of their efficacies. Plant extracts exhibited either a biofilm disruption and/or inhibition of biofilm formation. Psidium cattleianum extract and Psidium guajava exhibited enhanced efficacy compared to Chlorhexidine and NaOCl, respectively. Allium sativum demonstrated comparable efficacy to NaOCl. Furthermore, the combination of Cymbopogon martinii essential oil and NaOCl was found to be more effective than either alone when tested on a multispecies biofilm. However, the other plant extracts, such as Mikania Sprengel, Salvadora persica, Camellia sinensis, and Vitis vinifera showed efficacy but were still inferior compared to the control group.

**Conclusions:**

Overall, the tested plant extracts demonstrated promising potential for combating *in vitro* endodontic biofilms. In that context, integrating conventional therapy protocols with plant-inspired treatments may allow effective endodontic biofilm eradication. Hence, future research should focus on optimizing the synergistic combinations of these extracts with NaOCl to maximize the therapeutic outcomes. Heterogeneity amongst the studies prevented a meta-analysis.

## 1 Introduction

Endodontic infection is a polymicrobial infection currently classified as a “Biofilm-mediated disease” (1). Endodontic biofilms are usually made up of microorganisms embedded in an exopolymer matrix that can vary in composition and biology (2). This biofilm acts as a reservoir of chronic infections and a barrier to antimicrobial agents (3, 4). The common cause of endodontic treatment failure is often linked to residual micro-organisms in the root canal system, resulting from unsuccessful eradication of the biofilm (5). In some cases, this may lead to tooth extraction (6). Therefore, the major purpose of root canal treatment is to disturb the biofilm, reduce the bacterial load, and prevent microbial recolonization of the treated canal (7).

Root canal irrigation is considered a paramount step during endodontic treatment. Among multiple existing irrigants, sodium hypochlorite (NaOCl) is the most frequently used (8). Recent studies showed that NaOCl is utilized as the preferred irrigant among general dentists and endodontists in several countries, such as Australia, Turkey, and Spain (9–11). This preference is likely due to its potent antibacterial and antibiofilm properties as well as its ability to facilitate the dissolution of residual pulp tissue. However, a study conducted by Rosen et al. suggested that NaOCl might contribute to bacterial persistence by being extremely toxic to planktonic bacteria but failing to eradicate biofilm cells (12). Additionally, NaOCl can be highly toxic if accidentally extruded, and can cause allergic reactions in patients who are sensitive to chemical agents (13). Furthermore, it may reduce the mechanical properties of dentin and cause structural damage to its components, particularly collagen (14–16). Moreover, NaOCl can cause irritation to apical tissues, particularly in immature teeth with larger apices, where preserving the apical papilla is crucial for pulp regeneration therapies (17). Chlorhexidine (CHX) is another irrigant that has been proposed because of its antibacterial activity, substantivity, and lower tissue toxicity compared to NaOCl; however, it is unable to dissolve pulp tissue or efficiently eradicate biofilms (18, 19). Although, these synthetic chemicals proved efficacy in disinfection, the search for alternative irrigants continues, as no ‘ideal’ solution has yet been found. Consequently, in an attempt to avoid the limitations of these conventional synthetic irrigants, the search for potential natural adjuvants has been encouraged (20, 21).

Natural products have already been used in endodontics. For example, clove extract is regularly used as a common root canal sealer called zinc oxide eugenol (20), eucalyptol and orange extracts are also commonly used in clinics as solvents of the gutta percha for endodontic retreatment (22), also soybean and olive oil are increasingly proposed as lubricants for the removal of fractured instruments from root canals (23). Recent systematic reviews have highlighted the antimicrobial properties of plant extracts as root canal irrigants (24, 25). However, these studies did not fully address the antibiofilm efficacy of plant-based irrigants. To the best of our knowledge, there had been no previous systematic review addressing specifically the antibiofilm potential of plant extracts in combating root canal biofilms.

Hence, this systematic review provides a critical presentation of the antibiofilm effects of various plant extracts against *in vitro* single and multispecies biofilms compared to conventional root canal irrigants. This review also intended to explore the mechanism of action of these natural extract on biofilms, either by biofilm disruption or inhibition of biofilm formation.

## 2 Materials and methods

This systematic review was conducted in accordance with the Preferred Reporting Items for Systematic Reviews and Meta-Analyses (PRISMA) statement checklist 2020 (26).

### 2.1 Research question

The proposed research question for this study was “How effective are plant-based irrigation solutions in terms of antibiofilm activity, compared with conventional irrigation solutions?”

### 2.2 PICO elements

The research question was constructed based on the following PICO(S) schema including:

Population (P): Which is the plant extracts tested to be used as root canal irrigant, including essential oils, hydrolats, organic solvent-based extracts and pure plant extracts.
Interventions (I): Which is the antibiofilm activity of the plant extracts either by prevention of the biofilm formation, or biofilm disruption.
Comparators (C): Any conventional root canal irrigation solution (i.e., sodium hypochlorite and chlorhexidine).
Outcomes (O): The primary outcome of this review was the efficacy of plant extracts against endodontic biofilms either by biofilm prevention or eradication (assessment applying qualitative or quantitative criteria).
Study design (S): Experimental studies (e.g., laboratory-based studies, clinical trials), observational studies and, comparative studies if available.

### 2.3 Search strategy

Databases were searched according to the PICO using a combination of controlled vocabulary (MeSH terms) and free text terms. PubMed, Scopus, Web of Science, and EMBASE were explored from the oldest available data until the end of April 2024. The search equations were adapted according to the databases used (Table 1).

**Table 1:** Search strategy.

PubMed	((“Plant Extracts”[Mesh]) AND “Biofilms”[Mesh]) AND “Root Canal Irrigants”[Mesh]) ((“Plant Extracts”[Mesh]) AND “Biofilms”[Mesh]) AND “Endodontics”[Mesh]) ((“Plant Extracts”[Mesh]) AND “Biofilms”[Mesh]) AND (“Root Canal Irrigants”[Mesh] OR “Endodontics” [Mesh]) (“Plant Extracts”[Mesh] OR “Drugs, Chinese Herbal”[Mesh]) AND “Biofilms”[Mesh]) AND (“Root Canal Irrigants”[Mesh] OR “Endodontics” [Mesh])
Scopus	(TITLE-ABS-KEY (“root canal irrigants” AND “plant extracts” AND “biofilms”)) (TITLE-ABS-KEY (“plant extracts” AND “biofilms” AND “endodontics”)) (TITLE-ABS-KEY (“plant extracts” AND “biofilms” AND (“root canal irrigants” OR “endodontics”))) (TITLE-ABS-KEY ((“plant extracts” OR “drugs, Chinese herbal”) AND “biofilms” AND (“root canal irrigants” OR “endodontics”)))
Web of Science	ALL = (root canal irrigants AND plant extracts AND biofilms) ALL = (plant extracts AND biofilms AND endodontics) ALL = (plant extracts AND biofilms AND (root canal irrigants OR endodontics)) ALL = ((plant extracts OR natural compounds) AND biofilms AND (root canal irrigants OR endodontics))
Embase	(“root canal irrigants”/exp OR “root canal irrigants”) AND (“plant extracts”/exp OR “plant extracts”) AND (“biofilm”/exp OR “biofilm”) AND “endodontics” (“biofilms” AND (“root canal irrigants” OR “plant extract”/exp OR “plant extract” OR “natural compounds”) AND (“biofilm”/exp OR “biofilm”) AND “root canal irrigant” AND “endodontic”

### 2.4 Selection criteria

*In vitro* and *ex vivo* studies using at least one plant extract and one conventional root canal irrigant were included. Articles not published in English, studies in which plant extracts were used as intracanal medication, review articles, and studies focusing only on the effect of plant extracts against endodontic microorganisms in their planktonic state were excluded.

### 2.5 Data extraction

Articles were independently searched and screened by two authors based on the inclusion and exclusion criteria. After evaluation of titles and abstracts, duplicates were removed, and potential studies fulfilling the inclusion criteria were chosen for this review. The screening process and data extraction were performed using a customized reading grid in Microsoft Word (Microsoft Word Version 2310). The following data were extracted: (i) characteristics of the plant-based extracts used, including the plant of origin, concentration, volume, type of extract, and method of administration. (ii) characteristics of the experimental approaches, including models used for the study, biofilm type, composition, and the evaluation methods.

### 2.6 Risk of bias assessment

Two independent authors assessed the risk of bias of individual studies. The Joanna Briggs Institute (J.B.I.) checklist for risk of bias assessment was modified according to *in vitro* studies, following the evaluation framework available in the study by Teja et al. (27). The included studies were evaluated for the reporting of the experimental data, standardization of the protocol, and blinding. The risk of bias was scored as “ Low” when the details of the parameters without ambiguity, as “Unclear” when there was ambiguity. When no details were provided, it was scored as “High”.

## 3 Results

**Figure 1:**
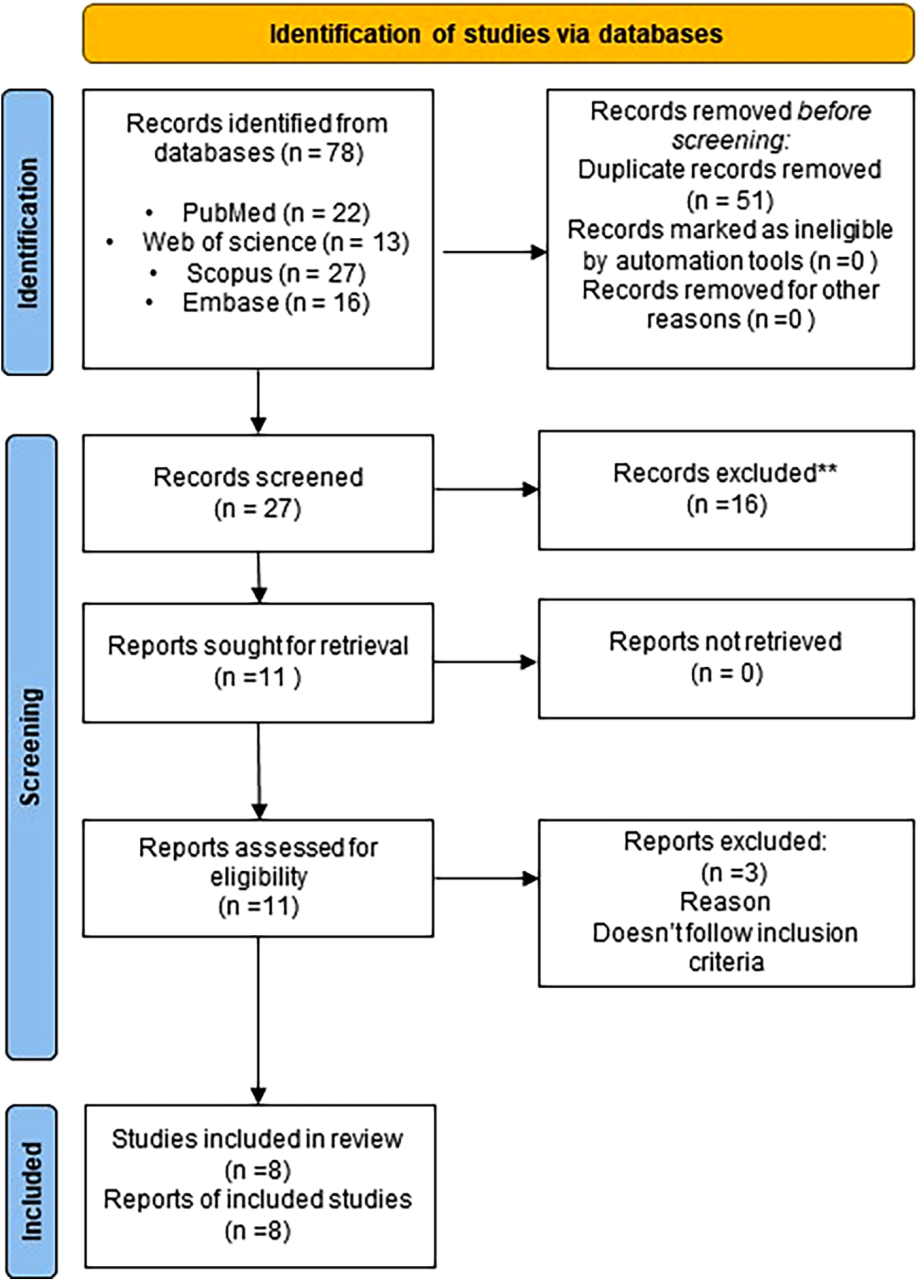
PRISMA 2020 flow diagram illustrating the outcome of the electronic database search.

A total of 78 studies were obtained after a search in four databases, including PubMed, Scopus, Web of Science, and Embase. After removing duplicate records, the titles and abstracts were screened. Eleven papers were included in the full-text review. Three papers were excluded because they did not meet the inclusion criteria (28–30). Eight studies were considered eligible and were included in the final review. A summary of the study selection procedure is presented in a flowchart, in accordance with PRISMA guidelines (Figure 1).

### 3.1 Plant extracts

Twelve (12) extracts from ten (10) plants including Mikania glomerata Sprengel (31), Salvadora persica (32), Psidium cattleianum (33), Cymbopogon martinii (34), Thymus zygis (34), Vitis vinifera (35), Triphala a herbal mixture of three plants Terminalia bellerica, Terminalia chebula, and Emblica officinalis (36), Camellia sinensis (Green Tea Polyphenols) (36), Psidium guajava (37) and Allium sativum (38) were described. The extracts were administered in various forms, including pure extracts (38), aqueous extracts (33), ethanolic extracts (32, 33), and essential oils (EOs) (34). Furthermore, some plant extracts were diluted in dichloromethane (DCM) (31) or dimethyl sulfoxide (DMSO) (35, 36), whereas others were delivered in the form of nanoparticles (37) (Table 2).

**Table 2:** Overview of the detailed characteristics of the representative plant extracts and controls.

Author and year	Name of the plant	Plant extract	Positive control	Negative control	Other irrigants
Martins et al. 2018 (31)	*Mikania glomerata Sprengel*	Dichloromethane (DCM) extract Component 1: kaurenoic acid Component 2: sodium salt derivative of kaurenoic acid (0.195 μg/ml to 0.4 mg/ml)	Chlorhexidine (0.115–59 μg/ml)	Not specified	–
Aljarbou et al. 2022 (32)	*Salvadora persica*	Ethanolic extract (10 mg/ml–10 μg/ml)	NaOCl (1% to 0,001%)	BHI	–
Prabhakar et al. 2010 (36)	*Triphala terminalia bellerica, Terminalia chebula, and Emblica officinalis* (Triphala) and *Camellia sinensis* (Green tea Polyphenols)	Plant powder in 10% dimethylsulfoxyde (DMSO) (60 mg/ml)	3 ml of NaOCl (5%)	3 ml NaCl	3 ml MTAD
Massunari et al. 2017 (33)	*Psidium cattleianum*	Groupe 1: Aqueous extract (PCAE) obtained by decoction in deionized water. Groupe 2: hydroethanolic extract (PCHE) (80 mg/ml)	Chlorhexidine (5× or 10× MLC)	Amphotericin B (5× or 10× MLC)	–
Marinković et al. 2020 (34)	*Cymbopogon martinii* and *Thymus zygis*	Essential oil dissolved in ethanol. *C. martinii* (2.5 mg/ml) *T. zygis* (10 mg/ml)	TAP Metronidazole 400 mg, Ciprofloxacin 200 mg and Minocycline 100 mg, mixed in the ratio of 1:1:1 dissolved in sterile distilled water.	NaCl	1st Protocol: 20 ml NaOCl (1,5%) 2nd protocol: 10 ml NaOCl (1,5%)
Fiallos et al. 2020 (35)	*Vitis vinifera*	6.5% grape seed extract dissolved in DMSO and 70% ethyl alcohol	2 ml NaOCl (5,25%)	NaCl	2 ml CHX (2%)
Birring et al. 2015 (38)	*Allium sativum*	Pure extract of raw garlic (GE) used at concentrations of 10%, 40%, and 70%	NaOCl (5,25%)	NaCl	–
Miglani and Tani-Ishii 2021 (37)	*Psidium guajava*	Selenium nanoparticles (SeNPs) derived from ethanolic extract of guava leaves. Group II: SeNPs (1 mg/ml)	500 μl NaOCl (5.25%)	Distilled water	500 μl CHX (2%) and 500 μl Ca(OH)2 (1 mg/ml)

### 3.2 Irrigant solutions were used as controls

Six studies used NaOCl as a positive control (32, 34–38) at concentrations varying between 0.001% (32) and 5.25% (35, 37, 38), whereas four studies used chlorhexidine (31, 33, 35, 37) (Table 2).

### 3.3 Microbial composition of the biofilms

Eight single species biofilms were described, using Enterococcus faecalis (32–38), Porphyromonas gingivalis (31), Parvimonas micra (31), Pseudomonas aeruginosa (32, 33), Actinomyces israelii (33), Candida albicans (33), Streptococcus mitis (34) and Streptococcus sanguinis (34). These biofilms were tested at different maturity ages, ranging from 24 h (33) to six weeks (36). A two-week multispecies biofilm composed of S. mitis, S. sanguinis, and E. faecalis was described in one study (34). In terms of the origin of the bacterial strains used to form the biofilms, reference strains were used in four studies (33, 35, 37, 38), clinical isolates in three studies (32, 34, 36), and one study combined both types of strains (31) (Table 3).

**Table 3:** Overview on the bacterial characteristics of the biofilms, administration protocol and duration in the included studies.

Author and year	Surfaces/Models used for the study	Studied biofilms	Biofilm age	Administration protocol	Volume	Time of contact
Martins et al. 2018 (31)	Microtiter plates	Single species biofilm of Porphyromonas gingivalis (ATCC 33277) and Clinical isolates of Parvimonas micra	24 h	No protocol specified	Not specified	72 h
Aljarbou et al. 2022 (32)	96-well microtiter plates	Single species biofilm of clinical isolates of Pseudomonas aeruginosa and Enterococcus faecalis	72 h	No protocol specified (Components were mixed)	Not specified	60 min
Prabhakar et al. 2010 (36)	Premolars	Single species biofilm of clinical isolates of Enterococcus faecalis	3 weeks and 6 weeks	No protocol specified (the samples in each group were immersed)	Not specified	Not specified
Massunari et al. 2017 (33)	96-well microtiter plates	Single species biofilm of Enterococcus faecalis (ATCC51299) Actinomyces israelii (ATCC 12102) Pseudomonas aeruginosa (ATCC 15442) Candida albicans (ATCC 26790)	Not specified	No protocol specified	200 μl	24 h
Marinković et al. 2020 (34)	96-well microtiter plates and Premolars	Single species and multispecies biofilm of clinical isolates of Streptococcus mitis, Streptococcus sanguinis, and Enterococcus faecalis	15 days	Protocol A: 20 ml of the essential oil based irrigant Protocol B: 10 ml NaOCl (1.5%) followed by 5 ml of the irrigant based essential oil and 5 ml NaCl	Protocol A: 20 ml Protocol B: 5 ml	10 min
Fiallos et al. 2020 (35)	24-well microtiter plates and Human Monoradicular root	Single species biofilm of Enterococcus faecalis (ATCC 29212)	21 days	No protocol specified (Dentinal disks were immersed in Grape seed extract)	2 ml	10 min
Birring et al. 2015 (38)	Flat-bottomed 96-well polystyrene microtiterplates	Single species biofilm of Enterococcus faecalis (ATCC 47077)	24 h, 1 week, and 3 weeks	No protocol specified (GE was immediately added to E. faecalis suspension to obtain 10%, 40%, and 70% concentrations)	Not specified	“Co treatment Group”: 24 h “24 h”, “1-week”, and “3-week” Groups: 10 min
Miglani and Tani-Ishii 2021 (37)	96-well microtiter plates	Single species biofilm of Enterococcus faecalis (MTCC 439)	48 h	No protocol specified (The cell culture was then treated with different test groups)	500 μl	48 h

### 3.4 Methods used for biofilm quantification

The minimum inhibitory biofilm concentration 50 (MICB 50) (31), tube dilution method (36), crystal violet method (32, 34, 37), and Anthone Bradford's tests (37) were described as quantitative approaches. The qualitative approaches involved confocal laser scanning microscopy (32, 35), “Live/dead” bacterial viability test (32), scanning electron microscopy (34) and fluorescence microscopy (38). In three studies, confocal laser scanning microscopy results and scanning electron microscopy results were converted into quantitative data by the authors (32, 34, 35) (Table 4).

**Table 4:** Overview of the experimental approaches and outcomes of assessment on the antibiofilm activity.

Author and Year	Antibiofilm activity assessed	Experimental approaches/Evaluation Method	Outcome	Plant extract VS Control group	Statistical Analysis performed
Martins et al. 2018 (31)	Inhibition of biofilm formation	Minimum Inhibitory Concentration Biofilm 50 (MICB50)	A biofilm reduction of both *P.gingivalis* and *P.micra* by Kaurenoic Acid and its sodium derivates.	*Mikania glomerata Sprengel* <CHX (0.115–59 μg/ml)	Not specified
Aljarbou et al. 2022 (32)	Inhibition of biofilm formation	Crystal Violet biofilm Assay and “Live/Dead” bacterial viability test and Confocal Laser Scanning Microscope	A biofilm reduction of *E.faecalis* biofilm and *P.aeruginosa* biofilm by *S.persica* extract at 10 mg/ml	*Salvadora persica* <NaOCl (1%)	One-way analysis et test *post hoc* de Tukey *p* < 0.05
Prabhakar et al. 2010 (36)	Inhibition of Biofilm formation and Biofilm disruption	Disc Dilution Method and Tube Dilution Method	A total elimination of *E.faecalis* biofilm with Triphala extract at the concentration of 60 mg/ml	Triphala and GTP <NaOCl (5%)	One-way analysis Student “*t*”test using SPSS software *p* < 0.05
Massunari et al. 2017 (33)	Biofilm disruption	Microtiter plate Assay	Total elimination of *E.faecalis* biofilm with aqueous extract of *P.cattleianum* at 10× MLC and its hydroethanolic extract at 5× MLC and 10× MLC. Total elimination of *P.aeruginosa* biofilm by hydroethanolic extract of *P.cattleianum* at 40 mg/ml. Only biofilm reduction of *A. israelii* by hydroethanolic extract of *P.cattleianum* at 2.5 mg/ml.	*Psidium cattleianum* >CHX (10× MLC)	Two-way ANOVA complemented by Tukey test
Marinković et al. 2020 (34)	Inhibition of Biofilm formation and Biofilm disruption	*In vivo*: crystal violet assay and Petri dish counting method. *Ex vivo*: Scanning Electron Microscopy	**Single species biofilms** Growth inhibition of *S.mitis* biofilm by both *C.martinii* (MIC, 2× MIC and 4× MIC) and *T.zygis* (MIC). No significant growth inhibition of *S.sanguinis* biofilm either by *C. martinii* or *T.zygis*. Biofilm reduction of *E. faecalis* by both *C. martinii* and *T. zygis* at 4× MIC.	*Cymbopogon martinii* +NaOCl (1,5%) >NaOCl (1,5%) alone	One-way ANOVA
			**Multispecies biofilm** Biofilm reduction by *C.martinii* at 4× MIC Growth inhibition by *T.zygis* at 10× MIC (protocol A) Biofilm reduction by *C.martinii* at 10× MIC (protocol B)		
Fiallos et al. 2020 (35)	Biofilm disruption	Laser Confocal Microscopy	Biofilm reduction of *E.faecalis* by *Vitis vinifera* grape seed extract at 6.5%	*Vitis vinifera* <NaOCl (5,25%)	Test de Shapiro–Wilk Tukey test ANOVA one way
Birring et al. 2015 (38)	Inhibition of Biofilm formation and Biofilm disruption	Fluorescence Microscopy and Microbial viability test	Both biofilm disruption and growth inhibition of *E.faecalis* biofilm by garlic extract at 70%	*Allium sativum* ≈NaOCl (5,25%)	ANOVA test using SPSS Version 16 *p* < 0.05
Miglani and Tani-Ishii 2021 (37)	Inhibition of Biofilm formation	Viable Cell Count Crystal Violet Antibiofilm Assay and Anthrone Bradford's tests	Growth inhibition of *E.faecalis* biofilm by SeNPs at 1 mg/ml	*Psidium guajava* (SeNPs) >NaOCl (5.25%)	ANOVA test using SPSS 24.0 and Student paired “*t*” test *p* < 0.05

### 3.5 Biofilm disruption vs. inhibition of biofilm formation

Three studies assessed the ability of plant extracts to inhibit biofilm formation (31, 32, 37). Two studies examined the ability of plant extracts to disturb already-formed biofilms (33, 35), and three studies evaluated the dual capability of the extracts to both disturb and inhibit biofilm formation (34, 36, 38) (Table 4).

### 3.6 Risk of bias assessment

**Figure 2:**
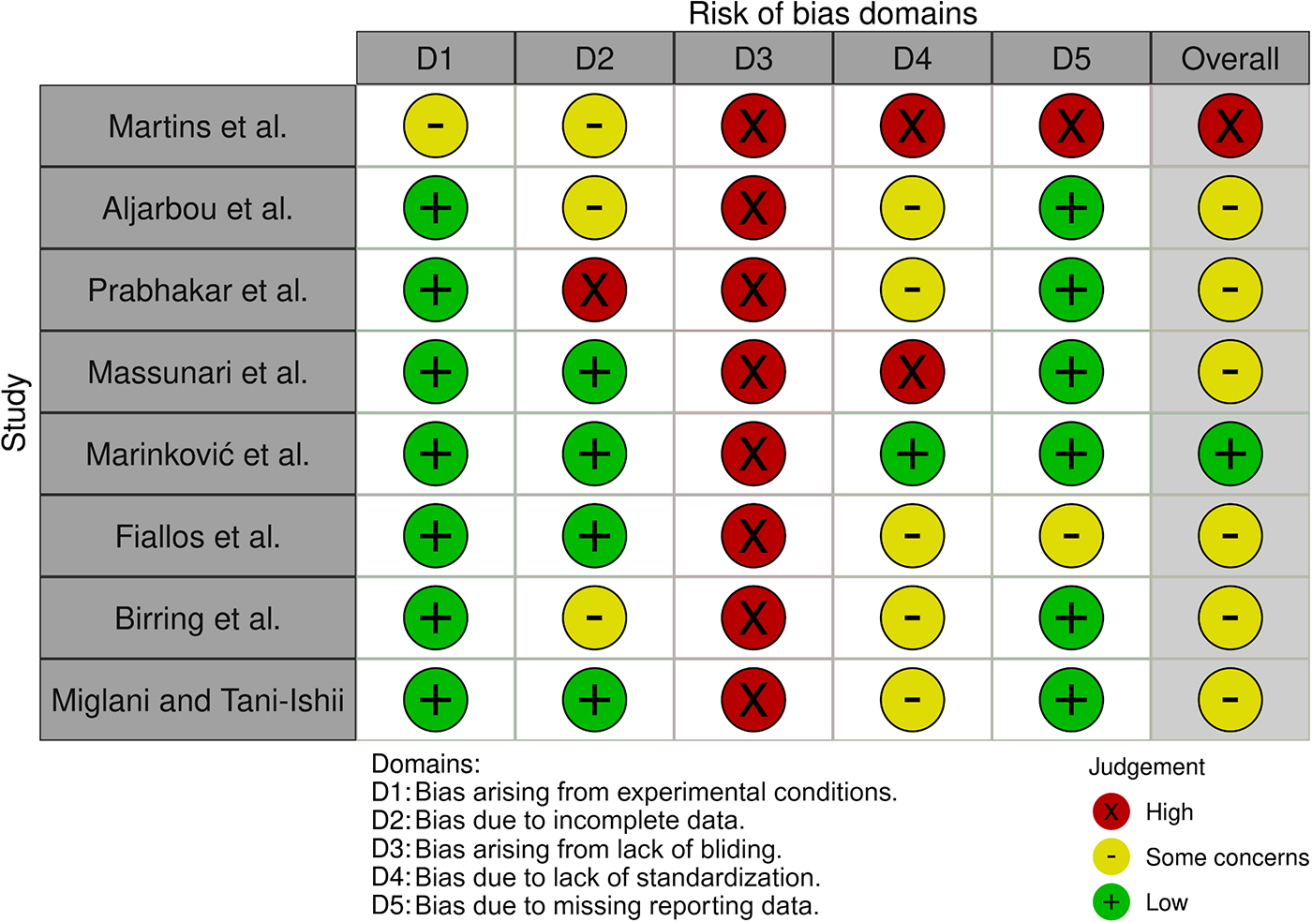
Evaluation of the risks of bias of the included studies.

Risk of bias assessment of the included studies was evaluated and shown in Table 5; Figure 2. Four studies did not disclose the volume of plant extract tested in their experiments (31, 32, 36, 38), whereas two studies did not report the number of experimental replications (31, 35). Moreover, one study did not report a negative control, and the statistical test employed (31).

**Table 5:** Detailed overview of risk of bias assessment of eligible studies.

Author and year	Experimental condition (Control groups, and type of extract)	Incomplete data (Time of contact & volume)	Blinding	Standardization	Reporting data (Replicates & statistical test)
Martins et al. 2018 (31)	Unclear (negative control not mentioned)	Unclear (Volume not mentioned)	High (not mentioned)	High (administration protocol not mentioned)	High (statistical test & replicates not mentioned)
Aljarbou et al. 2022 (32)	Low	Unclear (Volume not mentioned)	High (not mentioned)	Unclear	Low
Prabhakar et al. 2010 (36)	Low	High (Time of contact and volume not mentioned)	High (not mentioned)	Unclear	Low
Massunari et al. 2017 (33)	Low	Low	High (not mentioned)	High (administration protocol not mentioned)	Low
Marinković et al. 2020 (34)	Low	Low	High (not mentioned)	Low	Low
Fiallos et al. 2020 (35)	Low	Low	High (not mentioned)	Unclear	Unclear \(replicates not mentioned)
Birring et al. 2015 (38)	Low	Unclear (Volume not mentioned)	High (not mentioned)	Unclear	Low
Miglani and Tani-Ishii 2021 (37)	Low	Low	High (not mentioned)	Unclear	Low

## 4 Discussion

This systematic review evaluated the antibiofilm efficacy of 12 extracts, from ten plants against endodontic biofilms formed *in vitro* and *ex vivo*. The pooled data from eight studies revealed enhanced antibiofilm activity of the extracts obtained from Psidium cattleianum, Psidium guajava, Allium sativum, and Cymbopogon martinii compared to conventional root canal irrigants. However, a significant variation in the parameters related to the tested plant extracts, evaluated biofilms, and quantification methods was observed. Previous systematic reviews showed similar variability among the included articles when comparing herbal agents with sodium hypochlorite as root canal irrigants either in *ex vivo* studies (25) or in clinical and randomized controlled trials (24), but none of the antibiofilm parameters were investigated.

Concerning the plant extracts, the type of extracts and solvent seemed to play a determining role, since a total biofilm elimination was generally observed with the ethanolic extracts, while only biofilm reduction was induced with the aqueous extracts of the same plant at the same concentration. This was observed in our study with P. cattleianum extracts at a concentration of 5× MLC against a single-species biofilm of E. faecalis (33). Similar observations have been reported for other plants and biofilms. For instance, the ethanolic extract of Mangifera indica L showed higher efficacy than the aqueous extract in reducing Staphylococcus spp. biofilms (39). Moreover, the ethanolic extracts of Cytinus hypocistis and Cytinus ruber had not only the strongest antibacterial effect on Staphylococcus epidermidis but also total eradication, while the aqueous extracts showed only antibacterial effects at higher concentrations (40). Therefore, it can be proposed that the type of plant extract is strongly related to the resulting antibiofilm efficacy. In our study, different solvents were used for plant extract suspension. Prabhakar et al. (36) and Fiallos et al. (35) used DMSO for Triphala and V.vinifera extracts, while Marinković et al. (34) employed ethanol for C.martinii and T.zygis EOs to assess their antibiofilm effectiveness. These solvents possess inherent antimicrobial properties that could potentially bias the results (41). To address this, an independent evaluation of the solvent activity should have been included to determine an optimal concentration that dissolves the plant extract without significantly affecting tested microbial strains. Consequently, DMSO and ethanol should serve as controls. Otherwise, the absence of such controls may compromise the validity of findings, as antibiofilm efficacy might be partially attributed to the solvent. Additionally, in our study, Miglani and Tani-Ishii utilized guava leaves extract in the form of selenium nanoparticles. The outcomes showed superior antibiofilm efficacy compared to NaOCl at a concentration of 5.25% and CHX at a concentration of 2% (37). In fact, similar favorable outcomes have been observed in the literature when using nanotechnology-based delivery systems (42–44). Marinkovic et al. explored the potential of Cymbopogon citratus EO nanoemulsion as an auxiliary root canal disinfectant within the root canals of extracted teeth against E. faecalis biofilms. The results demonstrated the significant potential of the nanoemulsion form of EO as an irrigant compared with the use of the pure form of EO (45). These advantages may be attributed to the increase in surface area and enhanced bioactivity of natural compounds when used at the nanoscale (46–48).

**Figure 3:**
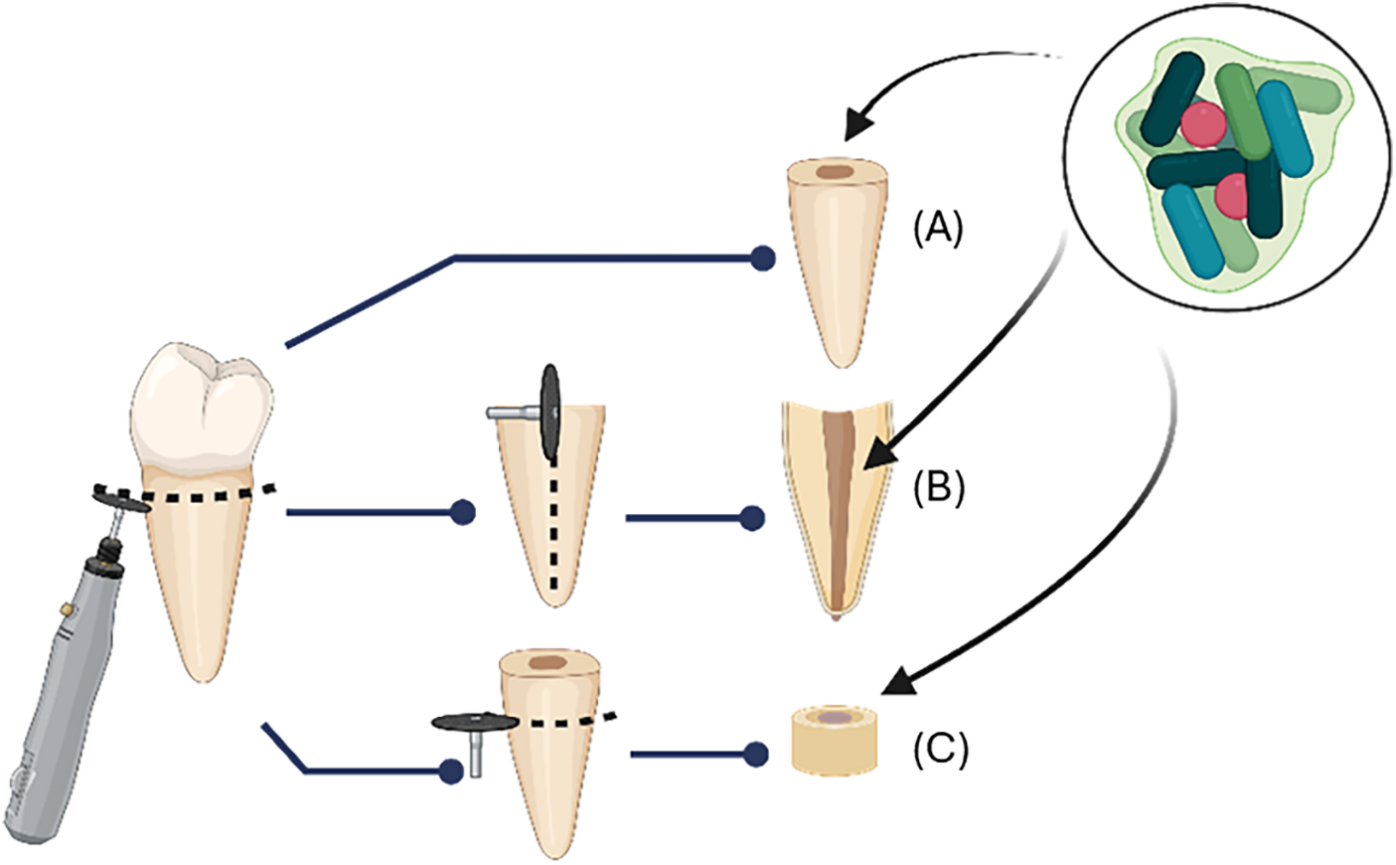
Illustration of the dental specimen used as substrate for biofilm growth. **(A)** Complete root; **(B)** Half of root; **(C)** Dentinal disks. Created with BioRender.com.

Several variations were also observed in the tested biofilms, including age, number of species in the biofilm, composition, origin, and the substratum used for biofilm cultivation. Regarding the age of the biofilm, our study confirmed that the bacteria in mature biofilms are more resistant to plant extracts than those in young biofilms. Prabhakar et al. showed that Triphala extract eliminated the biofilm completely at three weeks of growth, while only reducing it at six weeks of growth (36). Similar findings were reported for Streptococcus aureus and P. aeruginosa biofilms, as young biofilms of 24 h were more easily eradicated than older biofilms of 72 h, given that the biofilm matrix thickness increases with age (49, 50). Thus, it is essential to standardize the biofilm age when comparing the antibiofilm activity of different plant extracts due to the influence of biofilm age on biofilm biomass, thickness, cell count, and antibiofilm resistance. Stojicic suggested a three-week period of maturation when evaluating the antimicrobial activity of irrigants in *in vitro* or *ex vivo* experiments (51). In our study, the biofilms were composed of single species. Only one study described a multispecies biofilm (34). An *in vitro* or *ex vivo* biofilm model should be designed so that the findings can be extrapolated to clinical practice. A relatively simple model, such as a single species biofilm, may offer the advantage of simplicity and reproducibility; however, multispecies biofilms provide greater complexity, virulence, resistance, and more a more realistic representation of the biological systems in infected root canals. Therefore, it is recommended that future studies evaluate the antibiofilm effect of plant extracts *in vitro* using multispecies endodontic-like biofilm models to more closely simulate clinical reality (52). The included studies in this systematic review explored different bacteria typically found in primary or persistent root canal infections. E. faecalis was the most frequently tested species, either alone or in combination with other bacteria due to its popular pathogenicity in endodontology (53, 54). Only Massunari et al. tested a Candida albicans biofilm (33), as it is the most involved fungus in persistent and refractory root canal infections (55, 56). In the included studies, laboratory reference strains were mostly used to form biofilms, which offers high reproducibility because of their well-known genome sequences and properties. However, these strains present significant genetic and phenotypic differences compared to their clinical counterparts, primarily due to adaptative changes after being sub-cultured several times since their first isolation (57, 58). In contrast, three studies reported the use of wild clinical isolates (31, 33, 35) and one study used both types of strains (30). Furthermore, to study the antibiofilm efficacy of plant extracts, various substrate models have been used to grow biofilms. Polystyrene microtiter plates were used as synthetic surfaces (31–33). For natural surfaces, dental substrates have been described in three different forms: dentinal disks (35), complete roots (34), and half of roots (36) (Figure 3). While human dentin represents the natural environment of the biofilm, the extracted teeth used in these experiments usually originate from different individuals, introducing variability in dentin structure and composition (anatomy and age). This variability is not the case when nonbiological materials are used as substrates (52). However, the latter could alter the initial stage of biofilm formation since receptors on dentin are essential for bacterial adherence. To limit bias, standardization of the substrates could be proposed either for the synthetic ones using hydroxyapatite pretreatment or the use of comparable dental specimens, such as bovine root incisors (49).

Since biofilm removal is the preferred outcome in endodontic therapy, it is crucial to distinguish antimicrobial activity from antibiofilm activity. The antimicrobial activity could be considered a component of antibiofilm activity, as some plant extracts may exhibit potent activity against planktonic cells but little or no activity against biofilms (59, 60). For example, in our study, Prabhakar showed that extracts of Triphala and GTP exhibited similar antibacterial activity against E. faecalis strains with comparable MICs but showed different antibiofilm effects even at the same extract concentration and biofilm age (36). This difference in activity could be attributed to the presence of a polymeric matrix barrier in biofilms that could be selective according to the plant extract (2, 61) or to the presence of a specific compound in the plant extract with specific biofilm-related responses, resulting in a higher antibiofilm activity. These findings highlight the importance of considering not only antibacterial assays, but also antibiofilm assays when assessing the effectiveness of plant extracts. To assess the efficacy of plant extracts against biofilms, some studies have explored their potential in inhibiting biofilm formation, whereas others have focused on their ability to disturb established biofilms. It is important to evaluate the ability of plant-based root canal extracts to inhibit biofilm formation and disrupt biofilms to better understand their mechanism of action (60, 62). Furthermore, various quantitative and qualitative techniques have been employed to assess the antibiofilm effectiveness of different plant extracts, with culture and microscopy techniques being the most commonly used. However, it is recommended to use a combination of two or more complementary methods to draw conclusions about antimicrobial or antibiofilm effects (49, 63).

### 4.1 Qualitative review

Assessment of the antibiofilm efficacy of plant extracts against endodontic-like biofilms revealed that extracts of P. cattleianum (33) and P. guajava (SeNPs) (37) exhibited superior efficacy compared to chlorhexidine and NaOCl, respectively. A. sativum demonstrated comparable efficacy to NaOCl (38). Furthermore, the combination of C. martinii extract and NaOCl was found to be more effective than either alone (34). However, the other plant extracts, such as M. sprengel, S. persica, C. sinensis and V. vinifera showed lesser efficiency than the comparison group (31, 32, 35, 36). Hence, it is impossible to conclude on a favorable plant-based irrigant, as there were multiple variations in the included studies. However, this review thoroughly identified the key factors to be considered when evaluating the efficacy of plant extracts for root canal disinfection. Furthermore, it provides a comprehensive summary of the medicinal plants tested for antibiofilm activity for potential use as auxiliary root canal irrigants.

### 4.2 Quantitative review

Due to substantial methodological differences between the included studies, a meta-analysis could not be performed. The heterogeneity and variability of the collected data, such as the type of plant extract, biofilm models, and evaluation methods, made it difficult to conduct a meta-analysis.

## 5 Limitations

This systematic review disclosed various shortcomings and limitations in the studies investigating the effectiveness of plant extracts against endodontic biofilms. When employing the JBI checklist for risk of bias assessment, essential details, including the time of contact with the irrigant, the volumes utilized, and the appropriate controls were absent in several studies. Additionally, the findings of this systematic review were limited by the heterogeneity of the parameters employed, such as the type of extract, biofilm models, and the quantification methods. In addition, the overall quality of the studies reviewed was variable, with a limited number of high-quality studies meeting the inclusion criteria. Thus, this review emphasizes the importance of conducting well-designed studies and stresses the need for more rigorous protocols in future research.

## 6 Conclusions

This systematic review qualitatively synthesized eight heterogeneous studies to assess the antibiofilm effectiveness of plant extracts against single and multispecies biofilms. The study showed that while all plant extracts exhibited either a biofilm disruption and/or inhibition of biofilm formation, they were less effective compared to the control group except for P. cattleianum and P.guajava. The results described should be carefully analyzed and compared due to high variety in methodological parameters. This review emphasizes the importance to improve both on the reporting and the methodological aspects of the studies in order to elevate the level of certainty in evidence, thereby contributing significantly to clinical applications.

## Data Availability

The original contributions presented in the study are included in the article/Supplementary Material, further inquiries can be directed to the corresponding author.
